# A Modified Recombinant DNA-Based SARS-CoV-2 Vaccine Expressing Stabilized Uncleavable Spike Protein Elicits Humoral and Cellular Immunity against Various SARS-CoV-2 Variants of Concern

**DOI:** 10.1155/2023/5279979

**Published:** 2023-12-11

**Authors:** Rwaa H. Abdulal, Muhammad Yasir Khan, Najwa D. Aljehani, Zakiyah I. Fallata, Rahaf H. AlHarbi, Reem M. Alsulaiman, Ezdehar Abdulraouf Ghazal, Mohammad Basabrain, Mohammad A. Sanki, Magdah Ganash, Ishtiaq Qadri, Wesam H. Abdulaal, Mohammad W. Alrabia, Mazen Hassanain, Mohamed A. Alfaleh, Sathya N. Thulasi Raman, Levi Tamming, Tarfa Altorki, Turki S. Abujamel, Xuguang Li, Abdullah Algaissi, Rowa Y. Alhabbab, Anwar M. Hashem

**Affiliations:** ^1^Vaccines and Immunotherapy Unit, King Fahd Medical Research Center, King Abdulaziz University, Jeddah 21859, Saudi Arabia; ^2^Department of Biological Science, Faculty of Science, King Abdulaziz University, Jeddah 21589, Saudi Arabia; ^3^Biology/Microbiology Department, Faculty of Science, University of Jeddah, Jeddah, Saudi Arabia; ^4^Department of Biochemistry, Faculty of Science, King Abdulaziz University, Jeddah 21859, Saudi Arabia; ^5^Department of Clinical Microbiology and Immunology, Faculty of Medicine, King Abdulaziz University, Jeddah 21859, Saudi Arabia; ^6^Department of Surgery, Faculty of Medicine, King Saud University, Riyadh 11451, Saudi Arabia; ^7^Department of Pharmaceutics, Faculty of Pharmacy, King Abdulaziz University, Jeddah 21859, Saudi Arabia; ^8^Centre for Oncology and Regulatory Research, Biologic and Radiopharmaceutical Drugs Directorate, Health Products and Food Branch, Health Canada, World Health Organization Collaborating Center for Standardization and Evaluation of Biologicals, Ottawa, ON, Canada; ^9^Department of Biochemistry, Microbiology and Immunology, Faculty of Medicine, University of Ottawa, Ottawa, ON, Canada; ^10^Department of Medical Laboratory Sciences, Faculty of Applied Medical Sciences, King Abdulaziz University, Jeddah 21859, Saudi Arabia; ^11^Department of Medical Laboratories Technology, College of Applied Medical Sciences, Jazan University, Jazan 45142, Saudi Arabia

## Abstract

The appearance of several variants of concern (VOCs) of SARS-CoV-2 affects the efficacy of currently available vaccines and causes continuous spread and reinfection between humans. These variants possess different spike (S) protein mutations, which could affect viral pathogenicity, transmission, and immune escape. Herein, we develop a synthetic codon-optimized DNA vaccine (VIU-1007) expressing full-length S protein. The developed vaccine is stabilized by two K986P and V987P proline substitutions and resistant to cleavage by proteases such as furin by deletion of arginine residues (R682, R683, and R685) in multibasic furin cleavage site (RRAR). Additionally, it carries K417N, E484K, N501Y, and D614G substitutions in the receptor binding domain (RBD) derived from the beta VOC. Following the validation and characterization of the *in vitro* S protein expression, the humoral and cellular immunogenicity of VIU-1007 was assessed in immunized Balb/c mice. While both regimens elicited a Th-1-biased immune response based on S1-specific binding IgG isotypes, three vaccine doses significantly enhanced IgG levels. Furthermore, CD4^+^ and CD8^+^ memory T cell responses in spleens and draining inguinal lymph nodes were significantly higher in mice received three doses of VIU-1007 when compared to those received two doses only. Importantly, sera from mice immunized with three doses showed broad neutralization breadth against several SARS-CoV-2 variants, including alpha, beta, gamma, delta, and omicron VOCs. Moreover, the sera showed limited neutralization capacity against SARS-CoV-1, Bat SARS-like coronavirus WIV1, and MERS-CoV. Together, while these data suggest the presence of common neutralizing-rich epitopes between SARS-CoV-2 variants and some other betacoronaviruses, the ongoing evolution of SARS-CoV-2 could result in escape from vaccine-induced immunity, which requires a continuous update of vaccines.

## 1. Introduction

The unprecedented coronavirus disease-2019 (COVID-19) pandemic was initiated by the novel severe acute respiratory syndrome-coronavirus 2 (SARS-CoV-2) [1]. In the 21^st^ century, COVID-19 exemplifies the third extremely infective coronavirus introduced into humans, besides the SARS-CoV and the Middle East respiratory syndrome-coronavirus (MERS-CoV). Over the past 3 years, the pandemic has considerably impacted global health, with more than 675-million laboratory-confirmed cases resulting in 6.7-million deaths [2].

Developing effective therapies and preventive measures for COVID-19 have become a global priority to restrain the pandemic and overcome the challenges imposed by emerging variants. While several antiviral drugs and monoclonal antibodies have been tested or developed as countermeasures for COVID-19, vaccines have proven to be the most efficient strategy to control infectious diseases and decrease morbidity and mortality [1, 3]. To date, the development of vaccines against coronaviruses and SARS-CoV-2 has mainly focused on using the spike (S) protein as a target since it is the prime target for neutralizing antibodies (nAbs) due to its essential role in virus attachment and entry into the target host cells [4].

Several COVID-19 vaccine platforms have been employed worldwide, including viral vectors, mRNA, DNA, live attenuated viruses, or inactivated viruses [5–11]. Nucleic acid-based vaccines encode genetic instructions that rely on the host cell translational machinery to produce the protein antigen, allowing for greater antigen design flexibility [12]. The use of plasmid DNA constructs is the base of DNA vaccines, where a mammalian promoter drives the expression of a transgene encoding the protein of interest. DNA vaccines have several advantages over other systems, including thermal stability, the ability to provoke humoral as well as cellular immune responses, large-scale production in bacteria, and the capacity to encode multiple antigens in one vaccine [13].

The swift development of vaccines against SARS-CoV-2 is a tremendous achievement by the academia and pharmaceutical industry [14]. Throughout the pandemic, sequencing of SARS-CoV-2 isolates has shown the continuous evolution of the virus since its first emergence. While variants carry mutations throughout the genome, the S protein is one of the most heavily mutated viral proteins, particularly regions of the S recognized by nAbs [15–18]. Specifically, several mutations within the receptor binding domain (RBD) have emerged that reduce binding by antibodies [18]. Furthermore, SARS-CoV-2 evolution and emergence of variants resulted in immune escape and affected the efficacy of vaccines, which as a result, would require a continuous update [19, 20].

Although the S protein within the SARS-CoV and MERS-CoV is the perfect target for vaccine development, it lacks structural stability. It undergoes conformational changes that render neutralizing epitopes unexposed to the immune system [21–23]. Such studies have also demonstrated that replacing lysine and valine in positions 986 and 987, respectively, to prolines enhances spike stability and increases its expression levels [23]. Currently, at least five indorsed SARS-CoV-2 vaccines employ this prefusion-stabilizing tandem proline substitutions at positions 986 and 987 (K986P/V987P, known as S-2 P). Moreover, removing the multibasic cleavage site, which might support protein instability, has been found to stabilize the hemagglutinin (HA) of influenza viruses [24]. Recently, SARS-CoV-2 vaccines containing S protein with two proline substitutions with or without removed polybasic cleavage sites have increased vaccine immunogenicity. Moreover, they have been shown to provide better protection from virus challenges, with some displaying promising data in Phase 1 and 2 clinical trials [24–28].

Therefore, in this study, we sought to combine the most well-established approaches to date to stabilize the SARS-CoV-2 S along with selected mutations that appeared in most of the VOCs and have been shown to evade nAbs significantly. Thus, we produced and evaluated the immunogenicity of a synthetic DNA vaccine (denoted as VIU-1007) against SARS-CoV-2. The produced VIU-1007 vaccine bearing modified SARS-CoV-2 S protein possesses different mutations. These mutations include the two proline substitutions (K986P and V987P), the deletion of the three arginines R682, R683, and R685 from the furin cleavage site RRAR, and the introduction of K417N, E484K, N501Y, and D614G substitutions that appeared in the beta SARS-CoV-2 variant of concern (VOC).

## 2. Materials and Methods

### 2.1. DNA Construct

Mammalian vector (pcDNA 3.1) containing the ancestral Wuhan strain SARS-CoV-2 S protein codon-adjusted coding sequence was modified via site-directed mutagenesis utilizing Takara Bio purchased In-Fusion® HD Cloning Kit. In brief, In-Fusion PCR primers were designed using an online tool provided by the manufacturer (https://www.takarabio.com/learning-centers/cloning/primer-design-and-other-tools) to induce the following substitutions (K417N, E484K, N501Y, D614G, K986P, and V987P), and deletion of the three arginines R682, R683, and R685 from the furin cleavage site RRAR. The plasmid was linearized using an inverse PCR reaction as recommended by the manufacturer using a three-step PCR protocol and PrimeStar Max DNA Polymerase provided in the kit. PCR conditions were set for 35 cycles: 10 s at 98°C for denaturation, 15 s at 55°C for annealing, and 1.5 min at 72°C for the extension. Successful PCR amplification was verified by agarose gel electrophoresis. Gel-purified product was subjected to restriction digestion using the DpnI restriction enzyme provided in the kit and gel-purified using GenElute™ PCR Clean-Up Kit (Sigma-Aldrich, Burlington, MA). Then, an In-Fusion reaction of the purified product was performed according to the manufacturer's instructions. Circular pcDNA 3.1 plasmids were then transformed into DH5*α* competent cells, and the modified SARS-CoV-2 S gene was subcloned into mammalian expression vector pVAX1 (Invitrogen) as previously described [29, 30]. Restriction digestion and sequencing were finally used to construct confirmation.

### 2.2. Cells

Dulbecco's modified essential medium (DMEM), having penicillin, streptomycin, as well as a fetal bovine serum at 37°C in the presence of 5% CO_2_, was used to culture the human embryonic kidney 293 (HEK293) cells.

### 2.3. Western Blot

The transfection of HEK293 cells, 70%–75% confluent, with either VIU-1007 or control plasmid (2 *µ*g) was achieved using lipofectamine 2000 transfection reagent based on the company's guidelines. The cells were washed post-48 hr incubation at 37°C by cold PBS and lysed using radioimmunoprecipitation assay buffer. SDS-PAGE was next utilized to isolate the proteins obtained from the cell, then transferred on a polyvinyl difluoride (PVDF) membrane using Trans-Turbo Bio-Rad semi-dry transfer machine. Next, the S protein expression within the PVDF membrane was verified by performing a western blot using the in-house rabbit polyclonal anti-SARS-CoV-2 S protein antibodies (1 : 2,000) overnight at 4°C as previously described [29, 30], and 1 : 5,000 diluted anti-rabbit antibody conjugated to HRP. iBright FL1500 was utilized to detect the proteins.

### 2.4. Immunofluorescence

The transfection of HEK293 cells, 70%–75% confluent, with either VIU-1007 or control plasmid (0.5 *µ*g) was achieved using lipofectamine 2000 transfection reagent based on the producer's guidelines. The cells were then kept at 37°C for 24 hr. Postwashing the cells with PBS and removing the media, the cell fixation was done by incubating them for 10 min at 4°C with formaldehyde (4%). Postwashing the cells twice with PBS, PBS-Triton X-100 (0.2%) permeabilized the cells (at 4°C for 20 min). The cells were then blocked for 1 hr at room temperature after two additional washes, with a blocking buffer composed of goat serum (2%) in PBS-Triton-X-100. Postwashing the cells twice, they were stained with 1 : 2,000 diluted in-house rabbit polyclonal anti-SARS-CoV-2 S protein antibodies for 1 hr at 4°C in a humidified environment. Next, cells were incubated for 1 hr in the dark at room temperature with 1 : 500 diluted Alexa-Fluor 555 labeled anti-rabbit IgG H&L. Postwashing the cells three times with blocking buffer, DAPI stain with VECTASHIELD antifade mounting medium was used to mount the slide. Invitrogen™ EVOS™ M500 imaging system was utilized to capture the images.

### 2.5. Animal Studies

Female BALB/c mice aged 6–8 weeks were purchased from King Fahad Medical Research Center (KFMRC) animal house at King Abdulaziz University. All the work performed on the animal was done based on the guidelines of the Animal Care and Use Committee (ACUC) at KFMRC. The completed animal work was under the obtained ACUC approval (ACUC-21-10-44) and the ethical approval gained from the bioethical committee at KAU (approval number 04-CEGMR-Bioeth-2020). Moreover, all possible steps were taken to avoid animal suffering throughout each stage of this study. Mice were grouped into four sets (seven mice/set) and immunized intramuscularly with two or three doses of either VIU-1007 or control plasmid (100 *µ*g/mouse) every 21 days. Immunized mice blood samples were collected using retro-orbital bleeding 3 weeks post-second (Day 42) or -third dose (Day 63) to assess humoral responses. Spleens and inguinal lymph nodes were obtained from the immunized animals 5 weeks post-second (Day 56) or -third dose (Day 77) to evaluate the cellular immune responses. All mice were euthanized with 5% isoflurane.

### 2.6. Enzyme-Linked Immunosorbent Assay

ELISA was performed as published before [29, 30]. In brief, recombinant SARS-CoV-2 S1 subunit (1 *µ*g/ml) was used to coat 96-well maxibinding plates (SPL, Korea) overnight at 4°C. Postwashing the plates with PBS tween-20 (PBS-T), the plates were incubated for 2 hrs with 5% skim milk in PBS-T buffer (PBS-T/5%-milk) at room temperature to block the plates. Next, the washed plates were incubated for 1 hr at 37°C with twofold serially diluted mouse serum starting at 1 : 100. Then, 1 : 2,000 diluted rabbit anti-mouse IgG, IgG1, and IgG2 secondary antibodies conjugated to HRP were added to the washed plates and incubated for 1 hr at 37°C. Subsequently, the plates were washed, and TMB substrate was added. The reaction between the HRP conjugate and the TMB substrate was stopped using 0.16 M sulfuric acid. BioTek Synergy-2 microplate reader was utilized to enumerate the absorbance at 450 nm. Endpoint titers were represented as the reciprocals of the highest dilution with an OD value above 0.1 which was used as the cutoff OD value. The four-parameter logistic (4PL) curve in GraphPad Prism V9 software was used to compute the titers.

### 2.7. SARS-CoV-2 Pseudovirus Neutralization Assay

The SARS-CoV-2-pseudovirus neutralization assay used recombinant vesicular stomatitis virus- (rVSV-) based pseudovirus as published before [31]. rVSV expressing the ancestral Wuhan strain codon-optimized SARS-CoV-2 S protein full-length (rVSV-∆G/SARS-2-S ^*∗*^-Wuhan pseudovirus) or alpha, beta, gamma, delta, or omicron VOC, as well as SARS-CoV, Bat SARS-like coronavirus WIV1, and MERS-CoV pseudoviruses, were generated in BHK21/WI-2 cells transfected with pcDNA3.1 plasmids expressing corresponding proteins. Twenty-four hours postinfection, cells were infected with rVSV-G/G*∗*-luciferase, and the rVSV pseudoviruses were obtained from the supernatant. Collected pseudoviruses were titrated via determining luciferase activity in the infected E6 Vero cells. Titers were presented as relative luciferase units (RLU). Then, the neutralization assay was achieved by incubating an equal volume of twofold serially diluted heat-inactivated sera. Sera were prepared in DMEM with 5% FBS, and 1 : 20 was used as the starting dilution with DMEM containing 5 × 10^4^ RLU of each pseudovirus for 1 hr at 37°C. Subsequently, 100 *μ*l of the mixture was transferred to white 96-well plates holding confluent Vero E6 cells for 24 hr at 37°C. Each dilution of serum samples was tested in duplicate. After 24 hr, cells were lysed with 1x lysis buffer. Luciferase activity was determined through the addition of 50 *μ*l luciferase substrate (12.12 mg D-Luciferin in 40 ml dH_2_O) and 100 *μ*l luciferase buffer (1 mM ATP, 1 M MgSO_4_, 1 M DTT, and 1 M KPO_4_ dissolved into dH_2_O). BioTek Synergy-2 microplate reader was used to measure the luminescence activity. Each assay run included two controls, cell only (CC) and virus (VC). The following equation was used to calculate the luciferase-inhibited activity by every dilution of each sample: 100 – ((mean RLU from each dilution – mean RLU from CC)/(mean RLU from VC – mean RLU from CC)× 100). Then, the four-parameter logistic (4PL) curve in GraphPad Prism V9 software was used to compute the neutralization titers as half maximal inhibitory concentration (IC_50_).

### 2.8. Flow Cytometry

As previously described, splenocytes and inguinal lymph node cells were isolated and prepared from BALB/c mice [29, 30]. One million cells per well of splenocytes and lymph nodes were stimulated with a pool of 15-mer peptides overlapping by 11 residues (5 *μ*g/ml) covering SARS-CoV-2 S (GenScript) for 6 hr at 37°C. Stimulation was performed in the presence of 1 : 1,000 diluted Brefeldin A, added 1 hr after stimulation. For positive controls, cells were stimulated with ionomycin (1 *μ*M) and PMA (50 ng/ml) for 6 hr, while RPMI-1640 was used for the negative control cells. Postwashing, the cells were stained with a LIVE/DEAD™ Fixable Near-IR Dead Cell Stain Kit (Invitrogen, Carlsbad, CA) based on the producer's guidelines. Postwashing the cells, they were stained with surface antibodies against mouse CD8, CD4, CD44, and CD62L. After washing twice, Cytofix/Cytoperm Solution was used per the company's protocol to fix and permeabilize the cells. Cells were then intracellularly stained with anti-mouse IFN-*γ* and anti-mouse IL-2 antibodies for 30 min at 4°C. The BD FACSAria™ III flow cytometer was used to acquire the cells, and FlowJo v10 software was used to analyze the data. All flow cytometry antibodies were purchased from the BioLegend.

### 2.9. Statistical Analysis

Statistical analysis was produced by using GraphPad Prism V9 software. Statistical analysis was conducted using Mann–Whitney test. All values are represented as mean ± SD and statistical significance is reported as  ^*∗*^*p* ≤ 0.05,  ^*∗∗*^*p* ≤ 0.01,  ^*∗∗∗*^*p* ≤ 0.001, and  ^*∗∗∗∗*^*p* ≤ 0.0001.

## 3. Results

### 3.1. In Vitro Protein Expression from VIU-1007 DNA Vaccine Candidate

**Figure 1:**
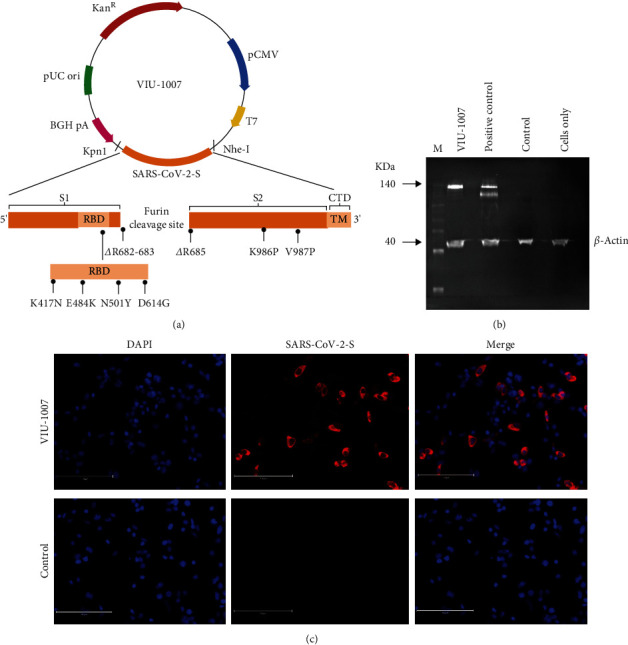
Vaccine design and *in vitro* protein expression of SARS-CoV-2 S protein. (a) Schematic representation of the constructed SARS-CoV-2 DNA vaccine, VIU-1007, after modification. Orange indicates the spike gene of SARS-CoV-2. (b) The modified S protein expression from transfected HEK293 cells with VIU-1007 construct is shown by western blot at the predicted size as a single band due to the removal of furin cleavage. In contrast, cells transfected with a plasmid expressing S protein from the ancestral Wuhan strain (positive control) showed two bands. No SARS-CoV-2 S protein bands were seen upon transfecting the cells with controls, plasmid or cells only. (c) VIU-1007 or control plasmid transfected cells immunofluorescent staining. Red represents transfected cells stained with anti-SARS-CoV-2 S rabbit polyclonal antibodies, while blue displays the nuclei counterstained with DAPI.

The candidate VIU-1007 plasmid DNA vaccine (Figure 1(a)) was constructed to express prefusion stabilized SARS-CoV-2 S by introducing two proline substitutions (K986P and V987P) in the hinge loop of the HR1 C-terminal as previously described [25–27]. Additionally, the three arginine residues (R682, R683, and R685) in multibasic furin cleavage site RRAR at the boundary between the S1/S2 subunits were deleted. Selected vital substitutions (K417N, E484K, N501Y, and D614G) in the RBD of the beta (B.1.351) VOC were also introduced. Next, using western blot, the expression of SARS-CoV-2 S protein from the candidate VIU-1007 vaccine was established in HEK293 cells. The results show a single protein band at the expected molecular weight under reducing conditions, unlike a plasmid encoding native ancestral spike containing the entire furin cleavage site, which produced two bands (Figure 1(b)). SARS-CoV-2 S protein expression was also visualized with immunofluorescence in transfected HEK293 cells.  Figure 1(c) shows that VIU-1007 transfected HEK293 cells had a robust immunofluorescent expressing S protein but not control plasmid transfected cells. These results indicate that the expressed antigen had retained its proper structural confirmation as it was recognized by antibodies generated against unmodified S protein from the ancestral Wuhan strain.

### 3.2. VIU-1007 Vaccinated Mice Induced Th1-Biased Humoral Immune Responses

**Figure 2:**
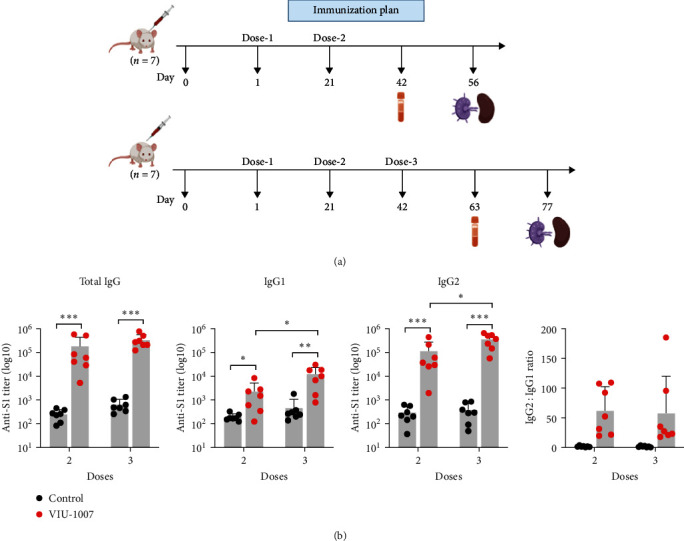
Humoral response in VIU-1007 immunized mice. (a) Representation of mice immunization plan. Mice were grouped into four sets (seven mice/set) and immunized intramuscularly with either two or three doses of VIU-1007 or control plasmid (100 *µ*g/mouse) every 21 days. Blood samples were withdrawn from immunized mice 3 weeks post-second (Day 42) or -third dose (Day 63) to assess humoral responses. (b) Mean endpoint titers of SARS-CoV-2 S1 specific binding total IgG, IgG1, and IgG2 as measured by ELISA after two and three doses are shown. IgG2 : IgG1 ratios are determined from samples collected after two or three doses of VIU-1007 or control plasmid. Data are shown as mean ± SD. Statistical significance is described as  ^*∗*^*p* ≤ 0.05,  ^*∗∗*^*p* ≤ 0.01, and  ^*∗∗∗*^*p* ≤ 0.001.

Next, the provoked immunogenicity by the VIU-1007 vaccine was examined. Anti-S1 IgG and its subtypes (IgG1 and IgG2) were measured in intramuscularly immunized Balb/c mice with 100 *µ*g/dose of VIU-1007 or control plasmid after two or three doses (Figure 2(a)). As shown in Figure 2(b), VIU-1007 elicited a strong total IgG response against SARS-CoV-2 S1 after two or three doses compared to the control group. Furthermore, S1-specific IgG1 and IgG2 levels were significantly induced in VIU-1007 immunized mice. While, no significant difference was observed when comparing the two regimens (two vs. three doses), S1-specific IgG1 and IgG2 were significantly higher in mice immunized with three doses than those received only two. Calculation of the IgG2 : IgG1 ratio showed higher levels of IgG2 to IgG1 by an average of >50 folds, suggesting a Th1-biased response (Figure 2(b)).

### 3.3. Mice Vaccinated with VIU-1007 Elicited Memory CD4^+^ and CD8^+^ T Cell Immune Response

**Figure 3:**
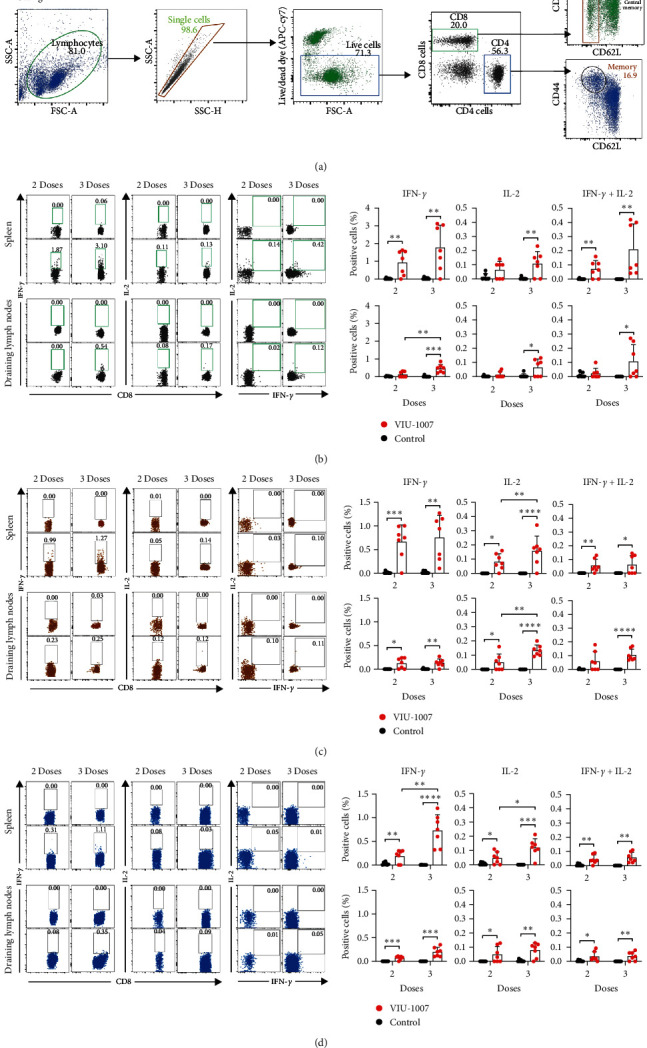
Cytokines response in CD4^+^ and CD8^+^ T cells from spleens and draining inguinal lymph nodes. Cells isolated from the spleens and draining inguinal lymph nodes were obtained from VIU-1007-immunized and control mice 5 weeks after receiving two or three doses. Cells were *ex vivo* restimulated with pooled peptides covering the complete S protein for 6 hr. After the first hour of culture, Brefeldin A was added. The cells were then incubated with live/dead viability and antibodies against CD4, CD8, CD62L, and CD44 (surface staining) and with antibodies for IFN-*γ* and IL-2 (intracellular staining). (a) Representative FACS plots of the gating strategies on live CD8^+^CD44^hi^CD62L^+^ central memory T cells, effector CD8^+^CD44^hi^CD62L^−^ memory T cells, and memory CD4^+^CD44^hi^CD62L^−^ T cells. Representative FACS plots (left) and histograms (right) displaying the percentages of IFN-*γ* and IL-2 expression by (b) central memory CD8^+^ T cells, (c) effector memory CD8^+^ T cells, and (d) memory CD4^+^ T cells. All the represented data were calculated by subtracting the values of the expressed cytokine by nonstimulated cells from the re-stimulated cells. Results are represented as mean ± SD from one experiment (*n* = 7). Statistical significance is reported as  ^*∗*^*p* ≤ 0.05,  ^*∗∗*^*p* ≤ 0.01,  ^*∗∗∗*^*p* ≤ 0.001, and  ^*∗∗∗∗*^*p* ≤ 0.0001.

Next, we investigated cytokine-expressing CD4^+^ and CD8^+^ memory T cells isolated from VIU-1007-vaccinated and control mice 5 weeks after receiving two or three doses. CD4^+^ and CD8^+^ T cells expressing IFN-*γ* and IL-2 in the spleens and the inguinal lymph nodes were evaluated by intracellular staining after stimulating the cells with an overlapping peptide pool covering the entire S protein. The gating strategies on the memory CD4^+^ and CD8^+^ T cells are shown in Figure 3(a). Following restimulation, central (CD44^hi^CD62L^+^) as well as effector (CD44^hi^CD62L^−^) memory CD8^+^ T cells expressing IFN-*γ* and IL-2 have significantly increased lymphocytes isolated from both the spleens and inguinal lymph nodes of VIU-1007 vaccinated mice relative to those isolated from control mice, particularly after the third dose (Figures 3(b) and 3(c)). Similar increases were seen in the percentages of IFN-*γ* and IL-2 double positive (DP) central memory CD8^+^ T cells (Figure 3(b)). However, although the DP effector CD8^+^ T cell frequencies were higher in VIP-1007 immunized mice compared to control mice, the increased level between the two dosing regimens was almost the same (Figure 3(c)). Following the third dose, lymphocytes from spleens and lymph nodes of VIU-1007 immunized mice displayed a significant increase in the proportions of IFN-*γ*^+^ and IL-2^+^ memory (CD44^hi^CD62L^−^) CD4^+^ T cells upon restimulation compared to control mice, characteristic of Th1 cytokine profile (Figure 3(d)). A slight but significant increase in the percentages of DP memory CD4^+^ T cells in the spleen and inguinal lymph nodes following VIU-1007 immunization was also observed (Figure 3(d)). These results demonstrate that immunized mice have acquired immunological T-cell memory in response to the VIU-1007 vaccine.

### 3.4. VIU-1007 Have Generated Cross-Neutralizing Antibodies against SARS-CoV-2 Variants

**Figure 4:**
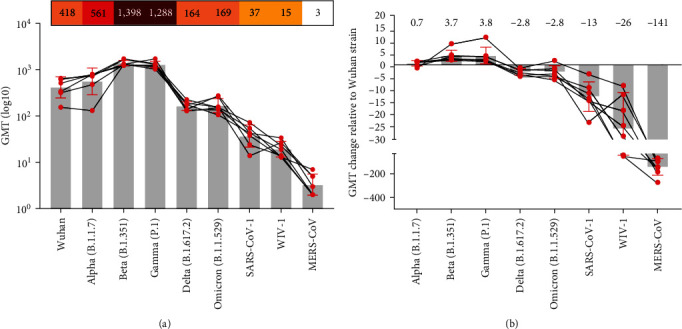
The median inhibitory concentration (IC_50_) of neutralizing antibodies (nAbs) induced by VIU-1007. The IC_50_ values of nAbs were determined against the different rVSV-*Δ*G-based pseudoviruses, including ancestral Wuhan strain and SARS-CoV-2 VOC alpha, beta, gamma, delta, and omicron as well as SARS-CoV, Bat SARS-like coronavirus WIV1, and MERS-CoV. Data are shown as mean ± SD. Titers are reported as geometric mean titers.

Having observed that three doses of VIU-1007 provoked higher levels of S1-binding IgG antibodies, we set to define the neutralization capacity of the induced antibodies against SARS-CoV-2 variants as well as other betacoronaviruses. As shown in Figure 4, serum samples were tested against pseudoviruses expressing S protein from ancestral Wuhan strain and alpha (B.1.1.7), beta (B.1.351), gamma (P.1), delta (B.1.617), and omicron (B.1.1.529) VOCs, as well as SARS-CoV-1, Bat SARS-like coronavirus WIV1 and MERS-CoV. While VIU-1007 induced strong nAbs against the ancestral Wuhan strain and alpha (B.1.1.7) variant, higher levels of nAbs were observed against beta (B.1.351) and gamma (P.1) VOCs (Figure 4(a)). This shift in the nAb response is likely because beta and gamma VOCs possess 4 and 3 of the amino acid substitutions introduced changes in VIU-1007 (i.e., K417N, E484K, N501Y, D614G in Beta VOC, and E484K, N501Y, and D614G in gamma VOC). While gamma VOC had K417T instead of K417N substitution, it seems to have little impact on the nAbs elicited by the vaccine. Albeit the lower levels of nAbs against delta (B.1.617) and omicron (B.1.1.529) VOCs and the existence of more mutations, especially in the omicron (B.1.1.529) VOC, cross-neutralization of these two variants suggests the presence of common neutralizing-rich epitopes between these variants. Despite the low levels, VIU-1007 also elicited some cross-nAbs against SARS-CoV-1 and Bat SARS-like coronavirus WIV1 but not MERS-CoV (Figure 4), suggesting potentially shared epitopes. As expected, no nAbs were detected in the sera obtained from mice vaccinated with the control plasmid (data not shown). Together, these data suggest that VIU-1007 can mostly elicit variant-specific nAbs, and continuous evolution of SARS-CoV-2 could result in escape from such nAbs.

## 4. Discussion

Since the beginning of the COVID-19 outbreak in late 2019, the continued evolution of SARS-CoV-2 has generated many different VOCs and variants of interest (VOIs), each having varying degrees of resistance to nAbs induced postvaccination or natural infection with SARS-CoV-2 [32–38]. To combat subsequent emerging variants, a vaccine that induces broad immune responses is needed [39]. As the S protein is an ideal vaccine target due to its roles in viral entry into target cells, a vaccine that combines some of the most successful approaches used to develop SARS-CoV-2 vaccines with introducing critical mutations in the RBD. These approaches appeared in the most aggressive variants, such as the beta VOC, which would help combat this virus's evolving nature.

In this study, we successfully generated a new DNA vaccine candidate against SARS-CoV-2, encoding a modified S protein. We also evaluated the immunogenicity produced by the developed vaccine in this study. The modified S protein possesses different mutations: substitution mutations (K417N, E484K, N501Y, D614G, K986P, and V987P) and deletion mutation of three arginines R682, R683, and R685 from the furin cleavage site RRAR. These modifications were intended to induce nAbs against VOCs in immunized animals. While 614G has become fixed in all the different VOCs that have emerged to date, K417N, E484K, and N501Y appeared together in the beta VOC and separate in the other variants and have been shown to have the most significant impact on escape from nAb responses [40, 41].

The residue N501 is located within the receptor binding motif (RBM) in the RBD [42, 43]. Because of its critical role in the interaction with the human receptor ACE2, it was suggested that N501Y substitution could increase RBD to ACE2 receptor when compared to the wild type [44, 45]. Interestingly, it was also suggested that it might contribute to maintaining the S protein in the so-called “open” prefusion conformation state, which can lead to stronger immune repones [46]. While it does not directly interact with ACE2 residues, K417T was associated with N501Y and has been suggested to have a significant role in evading humoral immune response [47]. Similar to residue 501, the amino acid at position 484 serves as a contact point for the interaction with ACE2 [48]. Thus, it was suggested that the E484K substitution increased the binding strength of the RBD and ACE2.

Post *in vitro* protein validation and characterization of our modified S protein that contains the above mentioned mutations, its immunogenicity was evaluated in Balb/c mice. Overall, the data indicated that VIU-1007 was capable of provoking robust humoral responses in mice following the third intramuscular dose as compared to the control group. Furthermore, our findings showed that VIU-1007 produced a Th1-biased protective immunity, described by antibody production of the IgG2 subclass compared to IgG1 and the increased secretion of Th1 cytokines (IFN-*γ* and IL-2) by memory CD4^+^ and CD8^+^ T cells in spleen and lymph nodes. Memory cells are the central component of the immune system against pathogens, including viruses. They can survive long-term and provide long-lasting protection upon reexposure to the same antigens through their capability of proliferating rapidly and acquiring effector function [49].

VIU-1007 elicited high levels of nAbs against beta and gamma VOCs as well as previously circulating alpha VOC and ancestral Wuhan strain. The levels of nAbs against beta and gamma VOCs were higher by ∼4 folds compared to alpha VOC and ancestral Wuhan strain (Figure 4). Although lacking K417N in gamma VOC might abrogate neutralization, VIU-1007 effectively neutralizes gamma pseudovirus. Levels lower by ∼3 folds against delta and omicron VOCs compared to ancestral Wuhan strain were also induced. This is consistent with findings from several recent studies showing substantial antibody evasion and reduced neutralization efficacy of the therapeutic monoclonal antibodies and sera from immunized people against SARS-CoV-2 omicron VOC and its subvariants [39, 50–52].

Low levels of cross-nAbs against SARS-CoV-1 and Bat SARS-like coronavirus WIV1 but not MERS-CoV pseudoviruses were also induced, similar to the levels elicited in individuals receiving COVID-19 mRNA vaccines [40]. Such antibodies might have been generated against the S2 domain of the S protein in SARS-CoV-1 and Bat SARS-like coronavirus WIV1 as it shows ∼90% homology with SARS-CoV-2 compared to the S1 domain, which shows ∼65% homology only. On the other hand, the inability to neutralize MERS-CoV by VIU-1007-induced sera is expected as its homology with SARS-CoV-2 is very low (∼29%).

Taken together, by introducing the indicated changes, our modified S vaccine-induced broad neutralization breadth against several SARS-CoV-2 variants, including alpha, beta, gamma, delta, and omicron VOCs, with a slight shift in favor of variants containing these mutations. Our approach provides essential information on optimizing the current vaccines and combating the continuous evolution of SARS-CoV-2.

## 5. Conclusions

Here, we have generated a stable DNA-based vaccine for SARS-CoV-2 expressing the entire-length S protein. Moreover, the developed vaccine carries K417N, E484K, N501Y, and D614G substitutions in the RBD derived from the beta VOC. The vaccine presented in this study was able to induce Th-1-biased immune responses as well as significantly high CD4^+^ and CD8^+^ memory T cell responses in mice. Most importantly, the vaccine showed broad neutralization breadth against several SARS-CoV-2 variants, including alpha, beta, gamma, delta, and omicron VOCs, with limited neutralization capacity against SARS-CoV-1, Bat SARS-like coronavirus WIV1, and MERS-CoV.

## Data Availability

All relevant data are within the manuscript and available upon request from the corresponding authors.
